# Global impact of diet and temperature over aquaculture of *Octopus vulgaris* paralarvae from a transcriptomic approach

**DOI:** 10.1038/s41598-019-46492-2

**Published:** 2019-07-16

**Authors:** P. García-Fernández, M. Prado-Alvarez, M. Nande, D. Garcia de la serrana, C. Perales-Raya, E. Almansa, I. Varó, C. Gestal

**Affiliations:** 1 Marine Molecular Pathobiology Group, Institute of Marine Research (IIM-CSIC), Vigo, Spain; 2 Instituto Español de Oceanografía, Centro Oceanográfico de Vigo, Vigo, Spain; 3 Serra Húnter Fellow, Department of Cell Biology, Physiology and Immunology, University of Barcelona, Barcelona, Spain; 4 Instituto Español de Oceanografía. Centro Oceanográfico de Canarias, Santa Cruz de Tenerife, Spain; 5 Instituto de Acuicultura Torre de la Sal (IATS-CSIC), Castellón, Spain

**Keywords:** RNA sequencing, Transcriptomics

## Abstract

Common octopus, *Octopus vulgaris*, is an economically important cephalopod species. However, its rearing under captivity is currently challenged by massive mortalities previous to their juvenile stage due to nutritional and environmental factors. Dissecting the genetic basis and regulatory mechanism behind this mortality requires genomic background knowledge. A transcriptomic sequencing of 10 dph octopus paralarvae from different experimental conditions was constructed via RNA-seq. A total of 613,767,530 raw reads were filtered and *de novo* assembled into 363,527 contigs of which 82,513 were annotated in UniProt carrying also their GO and KEGG information. Differential gene expression analysis was carried out on paralarvae reared under different diet regimes and temperatures, also including wild paralarvae. Genes related to lipid metabolism exhibited higher transcriptional levels in individuals whose diet includes crustacean zoeas, which had an impact over their development and immune response capability. High temperature induces acclimation processes at the time that increase metabolic demands and oxidative stress. Wild individuals show an expression profile unexpectedly similar to *Artemia* fed individuals. Proteomic results support the hypothesis revealed by transcriptional analysis. The comparative study of the *O*. *vulgaris* transcriptomic profiles allowed the identification of genes that deserve to be further studied as candidates for biomarkers of development and health. The results obtained here on the transcriptional variations of genes caused by diet and temperature will provide new perspectives in understanding the molecular mechanisms behind nutritional and temperature requirements of common octopus that will open new opportunities to deepen in paralarvae rearing requirements.

## Introduction

The common octopus (*Octopus vulgaris* Cuvier, 1797) is one of the most important cephalopod species worldwide due to its economic relevance (43,334 mT of global landings in 2014, FAO 2018; http://www.fao.org/fishery/statistics/en). In fact, *O*. *vulgaris* is a good candidate for aquaculture diversification mainly due to its fast growth rate, short life cycle, readily adaptation to captivity conditions, high food conversion efficiency and high economical value^1–3^. However, octopus culture has been achieved under captivity only few times due to the massive mortalities at the first stage (known as paralarva). The first successful studies were carried out in 1994 by Villanueva *et al*.^4^, passing the paralarval (planktonic) stage and obtaining benthic specimens. Later, in 2001 Iglesias and collaborators^5^ reared two specimens until their adulthood. However, these achievements were obtained using as live prey zoea of decapod crustaceans^5^, which is a commercially unviable feeding strategy. Therefore, most of the subsequent studies were focused on a more feasible preys such as brine shrimp *Artemia* which is the most common prey for the larval culture in marine species (mainly in finfish)^6^. Despite of the relative success of the *Artemia* in marine aquaculture, massive mortalities were observed in *O*. *vulgaris* paralarvae feeding with this prey in the range of 20–30 days post-hatching (dph).

Deficient nutritional factors seem to be one of the main causes behind the high mortalities in reared paralarvae^7,8^. In fact, *Artemia* presents several nutritional scarceness which could impede the development of paralarvae to settlement phase^8–10^. On the basis of this hypothesis, it seems necessary to find suitable enrichments for the *Artemia*, alternative live preys or even design inert diets that cover the nutritional requirements of the paralarvae. The improvement in knowledge about the physiology and health of the paralarvae under different diets could provide essential information to fulfill their nutritional requirements. Nutritional research is nowadays taking advantage of the “omics” technologies to find the effect of diet over physiology and health, to discover biologically active food components, to assess their quality and safety and to demonstrate their biological efficacy. Indeed the use of transcriptomic and proteomic studies has proven its capability to improve culture performance and quality of the product^11–14^. In addition to the diet, different culture conditions modulate the degree of energy consumption having an impact on growth and survival. Among them, temperature has shown critical effects over bivalve molluscs culture performance, especially at larval stage^15^. Even for well-known fish species temperature is an important parameter because of its impact over growth performance, welfare and health^16–18^. Indeed, recent studies carried on common octopus embryos have demonstrated that higher temperature induced a faster yolk consumption^19^. In contrast, the effect of temperature during post-hatching period has not been well studied yet, even when the most common temperature used in *O*. *vulgaris* paralarvae aquaculture is 20 ± 1 °C^7,20^ which is, in average, 4 ± 1 °C above the temperature of the seawater estimated during wild catches^21^.

Unfortunately, the impact of culture conditions over *O*. *vulgaris* paralarvae at metabolic and physiologic level is poorly or not understood. Moreover, up to date there is only one species of the family Cephalopoda with a complete assembled and annotated genome, *Octopus bimaculoides*^22^. In this context, the use of high-throughput technologies offers new and efficient strategies to perform a broad analysis at molecular level especially in non-model organism. Due to the lack of an annotated genome the use of an RNA-seq approach might improve the knowledge of their gene at sequences and expression level. There are only few studies focused on RNA-seq to evaluate the gene expression profiles in cephalopods and even less if we focused on the genus Octopus^23–27^. The combination of that approach with a proteome analyses can synergistically strengthen our understanding of common octopus paralarvae.

In the present study, transcriptomic analyses have been combined with shotgun proteomic SWATH-MS methodology (Sequential Window Acquisition of all Theoretical fragment ions followed by the mass spectrometry) to understand the impact of diet and temperature over the culture of *O*. *vulgaris* paralarvae. Comparative expression profiles of genes and proteins of octopus paralarvae reared in different conditions (diet or temperature) were performed. Moreover, a comparison study on wild and cultured octopus paralarvae was done. Regarding diet, cultured paralarvae fed with a suboptimal diet composed only by *Artemia* and a richer diet based on co-feeding of *Artemia* plus spider crab (*Maja brachydactyla*) zoeae were tested. To evaluate the effect of the temperature, we compared the effect of low temperature conditions (15 ± 1 °C), trying to simulate that found at their marine environment, and high temperature conditions (20 ± 1 °C) as one commonly used in octopus paralarvae aquaculture practices.

## Methods

### Paralarvae rearing and sampling

*O*. *vulgaris* paralarvae were obtained from a broodstock kept in captivity and reared following the conditions described by Moxica *et al*.^28^. Paralarvae were reared in black cylindrical 500 L tanks at an initial density of 6 individuals L^−1^. Two different seawater temperatures were used independently for rearing, low temperature (15 ± 1 °C) and high temperature (20 ± 1 °C), both at 35 psu salinity. Central aeration was used. Light intensity was of 300 lx with a 14 L:10D photoperiod. Two dietary treatments were tested: “diet A” consisted of paralarvae fed three times per day on *Artemia* metanauplii (Sep-Art EG, INVE Aquaculture, Belgium) enriched for 1 h with the microalgae *Nannochloropsis sp*. and *Isochrysis galbana* at 0.1 *Artemia* mL^−1^; and “diet Z” consisted on live crustacean zoea (*Maja brachydactyla*), dispensed three times per day at 0.01 zoeae mL^−1^ in co-feeding with *Artemia sp*, since it was not possible to obtain enough zoea to reach the same prey density of “diet A”. Samples were collected regularly over 15 days of culture.

Reared paralarvae were also compared to paralarvae captured from the environment. Wild paralarvae were collected around the Cíes Islands, Ría of Vigo (NW Spain). Surveys were carried out in 2015–2016 (n = 136) between May and September onboard the oceanographic vessel “José María Navaz” (Instituto Español de Oceanografía). During each survey, 5 trawls were performed along 2 transects of this area with a planktonic net of 2 m diameter and 500 μm mesh, to an average depth of 10 m over 10 min at a speed of 2 knots. Wild paralarvae were sorted on board from zooplankton samples using 30 L receptacles. Age estimation of wild paralarvae was conducted by counting parallel thin increments (known as rings) of the anterior region of the beak (upper jaw) following Perales *et al*.^21^. Once wild paralarvae age was estimated equally old culture paralarvae was selected for the comparative analyses.

Before any processing, animals were anaesthetized in diluted ethanol (2%)^29^, rinsed in distilled water and immediately euthanized by freezing in liquid nitrogen. Samples for RNA extraction and protein isolation were stored at −80 °C. Paralarvae dry weight was determined individually after oven drying for 24 h at 80 °C as described in Iglesias *et al*.^7^. The percentage of the standard growth rate (SGR %) for each trial were calculated following the formula: Standard growth rate (SGR; % BW·day^−1^) = (LnDW_f_ − LnDW_i_) · 100, where BW is the body weight, DW_f_ the 15 dph paralarvae dry weight and DW_i_ is the initial dry weight. All animal experiments were performed in compliance with the Spanish law 53/2013 within the framework of European Union directive on animal welfare (Directive 2010/63/EU) for the protection of animals employed for scientific purposes, following the Guidelines for the care and welfare of cephalopods proposed by Fiorito *et al*.^30^, and approved by the Ethic Committee of the National Competent Authority (project number: CEIBA2014-0108).

### Total RNA isolation and Illumina Sequencing

Total RNA was extracted from 10 dph whole paralarvae (3 samples per each experimental condition) using Trizol (ThermoFisher Scientific®) following the manufacturer instructions. The quality and integrity of total RNA was confirmed by both Safe-Red agarose gel and Agilent 2100 Bioanalyzer (Agilent®). Only RNA samples which show no degradation in the 18 S and 28 S rRNA fractions were further processed for sequencing (Centre for Genomic Regulation, CRG, Barcelona, Spain). Double-stranded cDNA libraries were prepared using the Kapa Stranded mRNA-seq kit (KK8420) following the manufacturer instructions with a mean library insert size of 201–300 bp. Briefly, RNA samples were first purified with two oligo-dT selection (poly(A) enrichment using oligodT beds), and then fragmented and reverse transcribed into double-stranded complementary cDNA. A total of 12 libraries were paired-ended sequenced (2 × 150 bp) in an Illumina HiSeq. 4000 platform from individual samples, including 3 biological replicates per group and 4 groups: Paralarvae fed with *Artemia* at low temperature (LA), paralarvae co-fed with Artemia and zoeae at low temperature (LZ), paralarvae co-fed with Artemia and zoeae at high temperature (HZ) and wild paralarvae (W). Raw reads were deposited in NCBI SRA with accession number PRJNA547720.

### Transcriptome assembly and functional annotation

Raw sequencing reads were assessed for quality using FASTQC v0.11.7 (http://www.bioinformatics.babraham.ac.uk/projects/fastqc/). The 12 RNA-Seq libraries were quality filtered using Cutadapt v1.14^31^ to remove Illumina adapters (TruSeqLT universal primer), sample indexes and low-quality reads (Phred cutoff score ≥ 30). The minimum read length cutoff was defined as 50 bp. The resulting reads were combined, assembled in a *de novo* transcriptome and quality reviewed using the resources of the Supercomputing Center of Galicia (CESGA; Santiago de Compostela, Spain) following the pipeline proposed by Mamrot *et al*.^32^. The minimum sequence length in the assembly was set 100 bp. The multiple assemblies output were collated in to non-redundant contigs by *tr2aacds* from the EvidentialGene^33^ package. The *tr2aacds* predicts amino acid sequences and transcript coding sequences, removes transcript redundancy based on coding potential, removes sequence fragments, clusters highly similar sequences together into loci, and classifies non-redundant transcripts as ‘primary’ or ‘alternative’. A summary of the assembly statistics (including back mapping rate) was obtained using Transrate v1.0.3^34^. “Bench-marking universal single-copy orthologs” (BUSCO) software v3^31^ was used to measure accuracy and completeness based in to the presence or absence of universal single copy orthologs common to Metazoa (a dataset consisting of 978 single-copy orthologous). Functional annotation was performed running NCBI BLAST algorithms v2.7.1^35^ in local mode on the subset of non-redundant contigs. The BLASTx algorithm^35^ was used to search against the SwissProt protein database (downloaded on February 2018 from UNIPROT) with a threshold E-value of 10^−5^. Transcripts which did not receive any hit against SwissProt database were researched against an “in lab” proteome database constructed by the combination of three proteomes: *O*. *bimaculoides*, *Crassostrea gigas* and *Homo sapiens* (downloaded on February 2018 from UNIPROT). The resulting annotations were mapped against the Gene Ontology (GO) and the Kyoto Encyclopedia of Genes and Genomes (KEGG) pathway databases using UniProt Retrieve/ID mapping tool. Annotation results were not manual reviewed.

### Analysis of differential gene expression (DGE) and GO enrichment analysis

To further identify annotated transcripts exhibiting significant differences in expression levels, pairwise comparisons were performed and the differentially expressed genes (DEGs) were extracted using the R package DESeq. 2^36^. Henceforth, we will refer to these annotated transcripts as “genes”. Diet effect was compared as follows: LA vs LZ, LA vs W, LZ vs W; and the effect of the temperature was studied trough LZ vs HZ contrast. The quantification was carried out running Kallisto^37^ over a data subset composed only by annotated contigs following Roncalli *et al*.^38^. The obtained pseudo counts were extracted with tximport^39^ in order to be used by DESeq. 2 in a gene-level mode (following Love *et al*.^40^). Significance was accepted only at a false discovery rate (FDR) cutoff of 0.05 and Log_2_ fold-change > 1. The GOs enrichment analysis in the subset of DEGs was then performed by pairwise comparison using a Fisher test following topGO v2.30.1^41^ R package.

### RNA-seq validation

To confirm DEGs obtained from the paralarvae transcriptomic profile, the gene expression level of 9 genes were quantified by RT-qPCR in a set of different samples (3 individual paralarvae) obtained under the same conditions and age. Sequences from the transcriptome assembly were used to design forward and reverse primers (Table 1) of the selected genes using PrimerQuest (https://eu.idtdna.com/PrimerQuest/Home/Index). The efficiency was determined following Pfaffl protocol (2001)^42^ and using five ten-fold dilution series. The extracted RNA of the selected samples was quantified using NanoDrop ND200 spectrophotometer (Thermo Scientific). First strand cDNA was synthesized using Maxima First Strand cDNA Synthesis kit for RT-PCR (Thermo Scientific) using 100 ng of total RNA, treated with DNAse (QIAGEN) to remove the remaining genomic DNA. RT-qPCR reactions were performed in a QuantStudio3 (Applied Biosystems) sequence detector. Each well contained 1 µL cDNA (dilution 1/10), 6.5 µL of SYBR green PCR master mix (Thermo Scientific), 4.5 µL of water and 0.5 µL of each diluted primer (10 mM). The final volume of each sample was 13 µL. The standard cycling conditions were: 95 °C for 10 min and then 40 cycles of 95 °C for 15 s and 60 °C for 1 min. Amplification of a single PCR product (a single sharp peak) from each experiment was monitored by a melting curve analysis. All reactions were performed as technical duplicates. The expression of the selected genes was normalized using the Ubiquitin as reference gene^43^ and analyzed following the Pfaff method^42^. Results were expressed as the mean ± standard deviation. A paired Student’s t-test, used to compare numerical data. Fold units were calculated by dividing the normalized expression values between conditions: LA vs LZ, LZ vs HZ and LZ vs W.

**Table 1:** Primer sequences used for RT-qPCR.

Gene	Primer	Sequence (5’ to 3’)	Gene description
Ubiquitin	**F:**	AGAAGGTTAAGTTGGCGGTTTTG	small protein present in all eukaryotic cells which target other proteins to promote their degradation, transport or regulate its interactions (act as reference gene)
	**R:**	CCAGCTCCACATTCCTCGTT	
HMOX1	**F:**	GAGCAATCGAGAAGGATCTG	essential enzyme in heme catabolism, cleaves heme to form biliverdin
	**R:**	TCTGGCCACGTATTTAGTTG	
Ferritin	**F:**	TATTTCAACTGCTCTCAGTCC	store iron in a non-toxic form
	**R:**	GGAACATGCTGAGAAGTTTATG	
PNLIPRP1	**F:**	CTCCCGAATATCTGGTTTGG	inhibitor of dietary triglyceride digestion
	**R:**	ACCAGTGTCTGTGTGAATG	
COX	**F:**	TGACTTAACATTGCGAAGGG	terminal enzyme of the mitochondrial respiratory chain
	**R:**	CACATTCACCAAATGTCTGAAC	
HMGCS1	**F:**	ATAATGCACCAATCACTTGC	condenses acetyl-CoA with acetoacetyl-CoA to form HMG-CoA, part of the synthesis route of (R)-mevalonate
	**R:**	ATTGTCCTGGGTGATGTG	
HNF4A	**F:**	TGATCCAGGTGCCAGAG	controls the expression of several genes which regulates the expression of several hepatic genes
	**R:**	AAATCGACCACGGCATTC	
NPC2	**F:**	CTACTGCAAGGACGAAACTAC	act regulating the transport of cholesterol through the late endosomal/lysosomal system
	**R:**	CTACTATCAGCCGGATTCTTG	
MOXD1	**F:**	GTACCTCGTTTCTTGGTAATTG	copper-dependent monooxygenase protein family
	**R:**	CCTGACAGGGAGAAAGATAAG	
DUOX	**F:**	CGATGAGACTTGGTGAATGG	plays a role in thyroid hormones synthesis and lactoperoxidase-mediated antimicrobial defense at the surface of mucosa
	**R:**	TGGTGATTACCGAATTTCCAG	

### Protein isolation

Proteins from whole individual paralarvae of 10 dph obtained from the same experimental groups analyzed at transcriptomic level were extracted in 100 µL Lysis buffer from 2D Grinding kit (GE Healthcare®) and quantified by RC_DC protein kit (BioRAD®). The protein profiles of the 24 samples (6 individuals for each of the 4 experimental groups) were evaluated with a 1D SDS PAGE gel. To prepare the library for the SWATH method 100 µg of total proteins extracts were mixed with Laemmli sample buffer 1X and denatured 5 min to 95 °C. Protein mix was loaded into a 1D SDS PAGE gel to visualize the protein profile and to eliminate contaminants. The gel was stained with colloidal Coomassie to visualize proteins. Each library career coming from the individual samples was excised into 4 fragments to increase protein identification and perform complete library of octopus paralarvae. Each gel fraction was excised and digested using sequencing grade trypsin (Promega®) following Shevchenko *et al*.^44^ to obtain the purified protein fraction. Protein digestion was stopped with 1% TFA (trifluoroacetic acid), followed by a double extraction with ACN (acetonitrile) and all peptide solutions were dried in a rotatory evaporator. Samples were resuspended with 2% of ACN and 0.1% TFA.

### SWATH LCMSMS analysis

Sample desalting was carried out on an analytical column (LC column, Nikkyo®) equilibrated in 5% ACN and 0.1% formic acid (FA) using 0.1% TFA. Peptide elution was carried out with a linear gradient of 5–35% ACN, 0.1% FA for 180 min at a flow rate of 300 nl/min. Peptides were analyzed in a mass spectrometer nano ESIqQTOF (5600 TripleTOF, ABSCIEX). TripleTOF was operated in information-dependent acquisition mode, in which a 250 msTOF MS scan from 350–1250 m/z was performed followed by 150 ms product ion scans from 350–1500 m/z on the 25 most intense 2–5 charged ions. The rolling collision energies equations were set for all ions as for 2+ ions. The mass spectrometry proteomics data have been deposited to the ProteomeXchange Consortium via the PRIDE partner repository with the dataset identifier PXD014141.

ProteinPilot default parameters were used to generate peak list directly from 5600 TripleTOF data and generate a list of 1,984 proteins which were identified with a FDR of 1% without taxonomy restriction using Expasy protein database. The search (identification) was conducted against an *O*. *vulgaris* paralarvae protein database previously generated (a total of 107,557 sequences obtained after translation to 6-reading frames, from the *de novo* transcriptome generated in this study, based on TransDecoder pipeline^45^).

The TripleTOF was operated in SWATH mode in which a 0.05 s TOF MS scan from 350–1250 m/z was performed, followed by 0.08 s product ion scan from 350–1250 m/z on the 32 defined windows (3.05 sec/cycle) of 15 Da. The data obtained from SWATH were analyzed by Peak View in order to obtain quantitative information which could be analyzed with Marker View. PCA was performed as preliminary analysis of samples.

The analysis of the data was performed using a multiple regression model with Elastic Net penalty by the use of the R library glmnet^46^. This restriction strategy made possible to use the model as a tool to perform variable selection (ie. the proteins identified by SWATH). The data were compared by diet and temperature as in DGE and GO analyses (see 2.4 for details).

## Results

### Culture

The initial average dry weight of hatchling set reared at environment temperature was 0.33 ± 0.02 mg, while it was 0.35 ± 0.02 mg for hatchling set fed on traditional reared method (high temperature). After 15 days of trial, the HZ paralarvae showed the highest increment of growth, reached a dry weight of 1.17 ± 0.19 mg with a standard growth rate of 6.003%. The LZ paralarvae showed a lower feed activity and achieved a dry weight of 0.56 ± 0.09 mg at 15 dph and a percent of SGR of 3.67% BW. The lowest percent of standard growth rate was of 0.75% BW to the LA paralarvae, showing a dry weight of 0.36 ± 0.05 mg at 15 dph and significantly differences (*p* < 0.05) among all trials (see Table 2). Both experiments were ended before 20 dph due to mortalities observed at late planktonic stages, before settlement, an event that is very common in the attempts of culture of this species^7,20^. Therefore, data concerning to final survival rate of the culture was not possible to obtain.

**Table 2:** Paralarvae culture.

Age (dph)	Temperature (°C)	Diet	DW (mg)	SGR (%)
0	low	—	0.325 ± 0.022	—
15	low	A	0.364 ± 0.050	0.751
15	low	Z	0.564 ± 0.091	3.666
0	high	—	0.351 ± 0.019	—
15	high	Z	1.166 ± 0.189	6.003

Summary of growth in terms of dry weight and SGR %.

### RNA sequencing and de novo assembly

**Figure 1:**
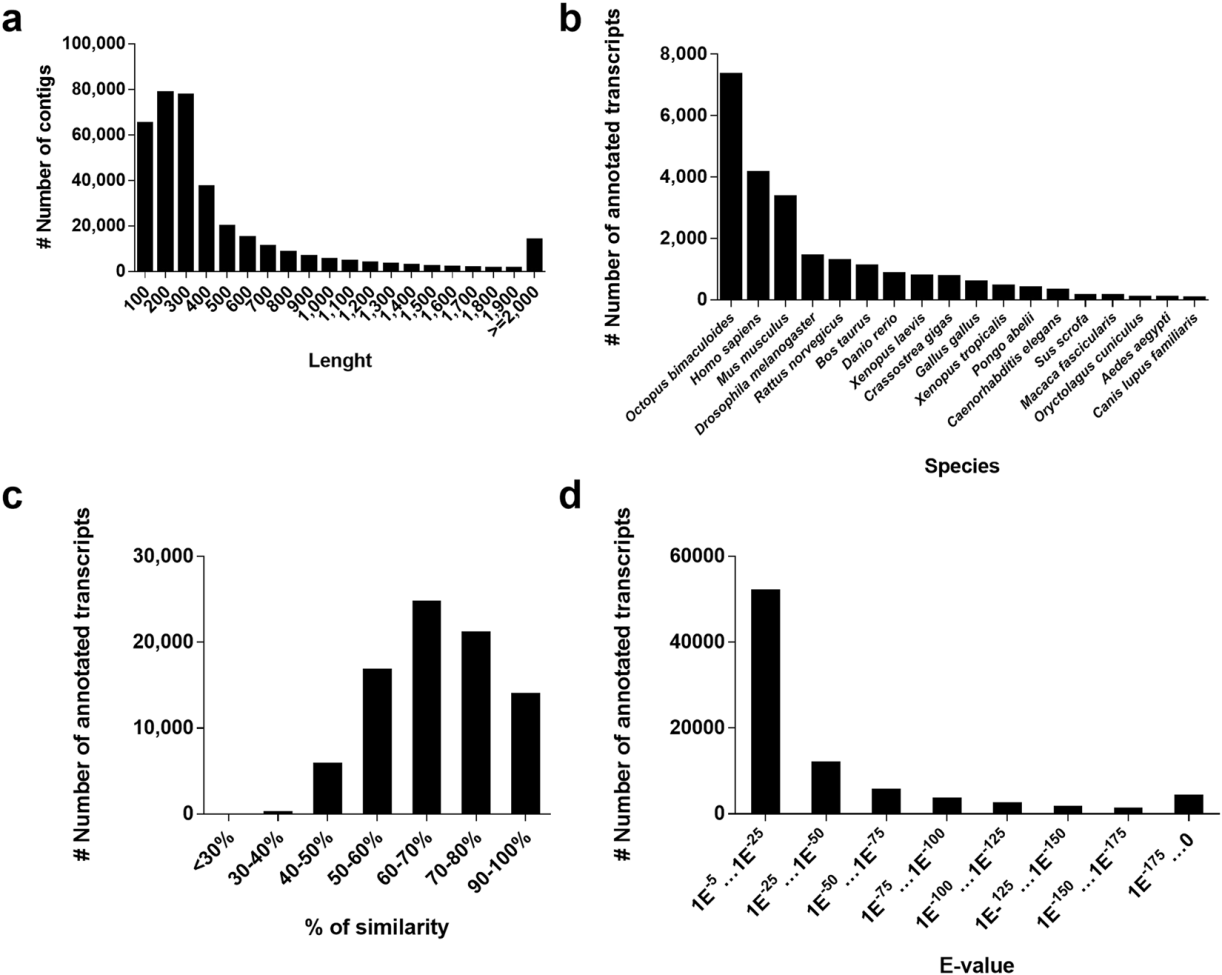
Annotation summary of *O*. *vulgaris* paralarvae transcriptome: (**A**) Length distribution of contigs in *O*. *vulgaris* paralarvae transcriptome. (**B**) Number of *O*. *vulgaris* paralarvae contigs annotated to different species. The hits against model organism carry the main functional information. Distribution of similarity (**C**) and e-value (**D**) of the annotated contigs. The cut-off in terms of e-value was 10E^−5^ and most of the hits were in the range of 10E^−5^ to 10E^−20^ and also below 10E^−100^. For the similarity rate, the cut-off was 30% and most of the hits were around 50–80%.

A total of 614 million (M) paired-end sequences were generated for the assembly of the *de novo* transcriptome (135 M, 162 M, 180 M and 137 M raw reads from LA, LZ, W and HZ groups, respectively). After filtering for adaptors and low quality reads a total of 577 M reads were retained (Table 3). Filtered reads were *de novo* assembled into 363,527 contigs. The proportion of unknown bases in the assembled contigs was 0% and the GC content of the final assembly was 38%. The smallest contig had a length of 100 bp and the longest 72,793 bp. The average length of the contigs were 531 bp (N50 = 937 bp) (Table 3). The 28.81% of contigs were longer than 500 bp, and 11.91% were longer than 1,000 bp (Fig. 1A).

**Table 3:** *De novo* assembly annotation statistics. Summary of the *O*. *vulgaris* transcriptome generated by RNA-seq de novo assembly.

Sequencing	
Raw reads (#)	613,767,530
**Assembly**	
Assembled contigs (#)	363,527
Average contig lenght (#)	531
Longest contig length (#)	72,793
Total length of all sequences in assembly (bp)	202,984,452
GC content (%)	38
Mapped reads (%)	78.55
**Annotation**	
Core Metazoa Genes (%)	99.7
Transcripts with BLAST hits (#)	82,513
Transcripts with GO terms (#)	58,618
Transcripts with KEGG terms (#)	30,654

### Functional annotation of contigs

BLASTX search with an E-value threshold at 1 × 10^−5^ reported 82,513 annotated transcripts with at least one hit after mapped against the selected databases (Table 3). 43.73% of these annotated transcripts (36,083) were mapped to SwissProt. 13.06% of these showed an E-value below 1 × 10^−100^, whereas remaining annotated transcripts (86.94%) showed E-values ranged between 1 × 10^−5^ and 1 × 10^−100^ (Fig. 1D). 19% of the sequences have a similarity greater than 80% and the remaining 81% of the sequences had a similarity ranging from 30% to 80% (Fig. 1C). Among the species distribution, the three species with the highest representation on Blast searches were *O*. *bimaculoides* (57%), *H*. *sapiens* (12%) and *M*. *musculus* (6.3%) (Fig. 1B). When the assembled contigs were annotated using the *O*. *bimaculoides* assembled genome, 79,580 contigs had a hit, however most of them correspond to uncharacterized proteins.

**Figure 2:**
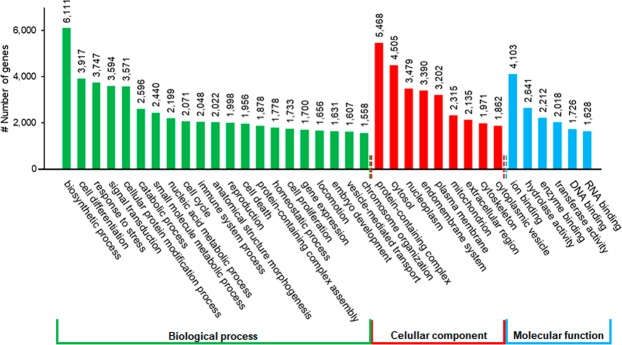
Functional classification of transcripts of *O*. *vulgaris* paralarvae transcriptome by GO terms.

Regarding categorization of the biological function, 71% of the mapped transcripts into UniProt databases also matched to at least one GO term (77.51% for biological process, 12.86% for molecular function and 9.63% for cellular component). At biological process category, the most abundant functional groups were “biosynthetic process” (GO:0009058), “cell differentiation” (GO:0030154) and “response to stress” (GO:0033554), among others (Fig. 2). At molecular function category, “ion binding” (GO:0043167), “hydrolase activity” (GO:0016787) and “enzyme binding” (GO:0019899) were the most highly represented functional groups, while the most frequent in cellular component category were “protein-containing complex” (GO:0032991), “cytosol” (GO:0005829), and “nucleoplasm” (GO:0005654), among others (Fig. 2).

**Figure 3:**
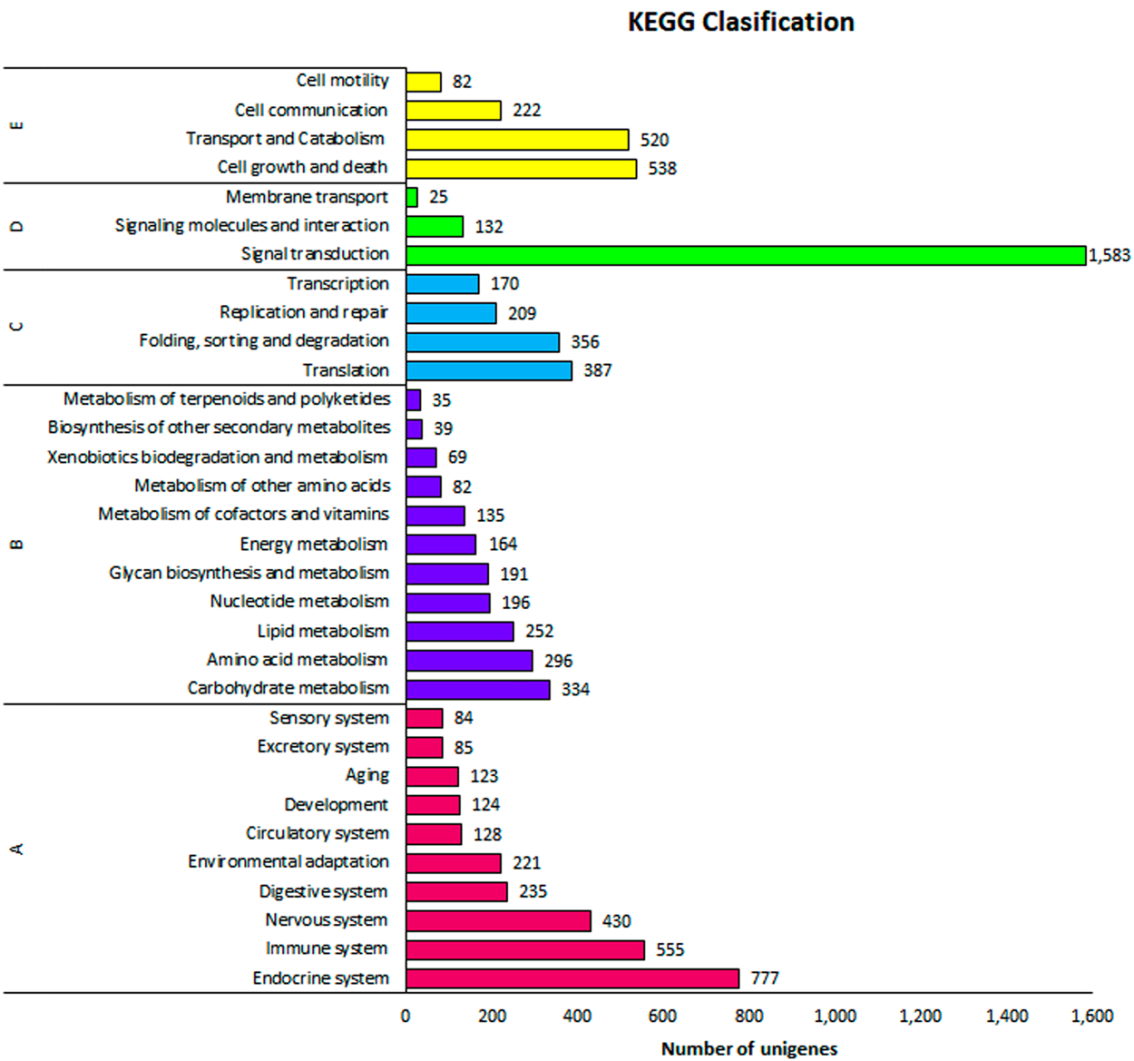
Functional classification of transcripts of *O*. *vulgaris* paralarvae transcriptome by KEGG terms. The colors represent the five KEGG sections. The numbers at the end of each bar indicate the numbers of genes in each category. Capital letters refers to KEGG main divisions: (**A**) organismal systems, (**B**) metabolism, (**C**) genetic information processing, (**D**) environmental information processing and (**E**) cellular process.

At least one KEGG identifier was obtained in 30,654 annotated transcripts (Table 3). Three main levels were observed concerning metabolic pathways (Fig. 3). Among genes related to carbohydrate and amino acid metabolism, a total of 41, 35, and 30 genes predicted to encode inositol phosphate metabolism, glycolysis/gluconeogenesis and pyruvate metabolism, respectively were highlight. Lipid metabolism, with genes related to glycerophospholipid (48 genes) and glycerolipid metabolism (31 genes) appeared also highly represented. In addition, other important lipid metabolism categories were found, i.e.: fatty acid degradation (25 genes), arachidonic acid metabolism (18 genes), fatty acid elongation (14 genes) and biosynthesis of unsaturated fatty acids (13 genes). Energy metabolism, nucleotide metabolism and glycan biosynthesis and metabolism were also found. Among the organismal system, genes related to endocrine system, immune system and nervous system were highly represented.

### Identification of DGE induced by diet or temperature

**Figure 4:**
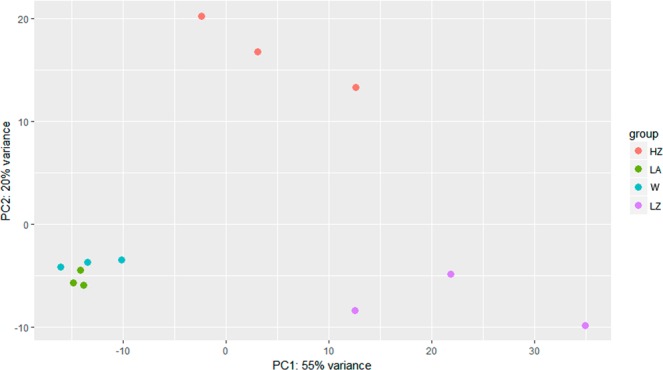
PCA plot of transcriptomic data of the *O*. *vulgaris* paralarvae individuals under study. Representation of the overall similarity, based on their transcriptomic, between the 12 sequenced individuals categorized in 4 groups: feed on *Artemia* (LA), feed on zoea (LZ), wild paralarvae (W) and high temperature (HZ).

The PCA plot (Fig. 4) showed the relationship at gene expression level between replicates and conditions. Individual samples from each group plot close to each other. A clear segregation of the different experimental groups, highlighting the existence of fundamental differences caused by diet and temperature was observed. Specially, principal component 2 (which summarize 22% of the variability) shows an important discrimination effect based on the temperature.

**Figure 5:**
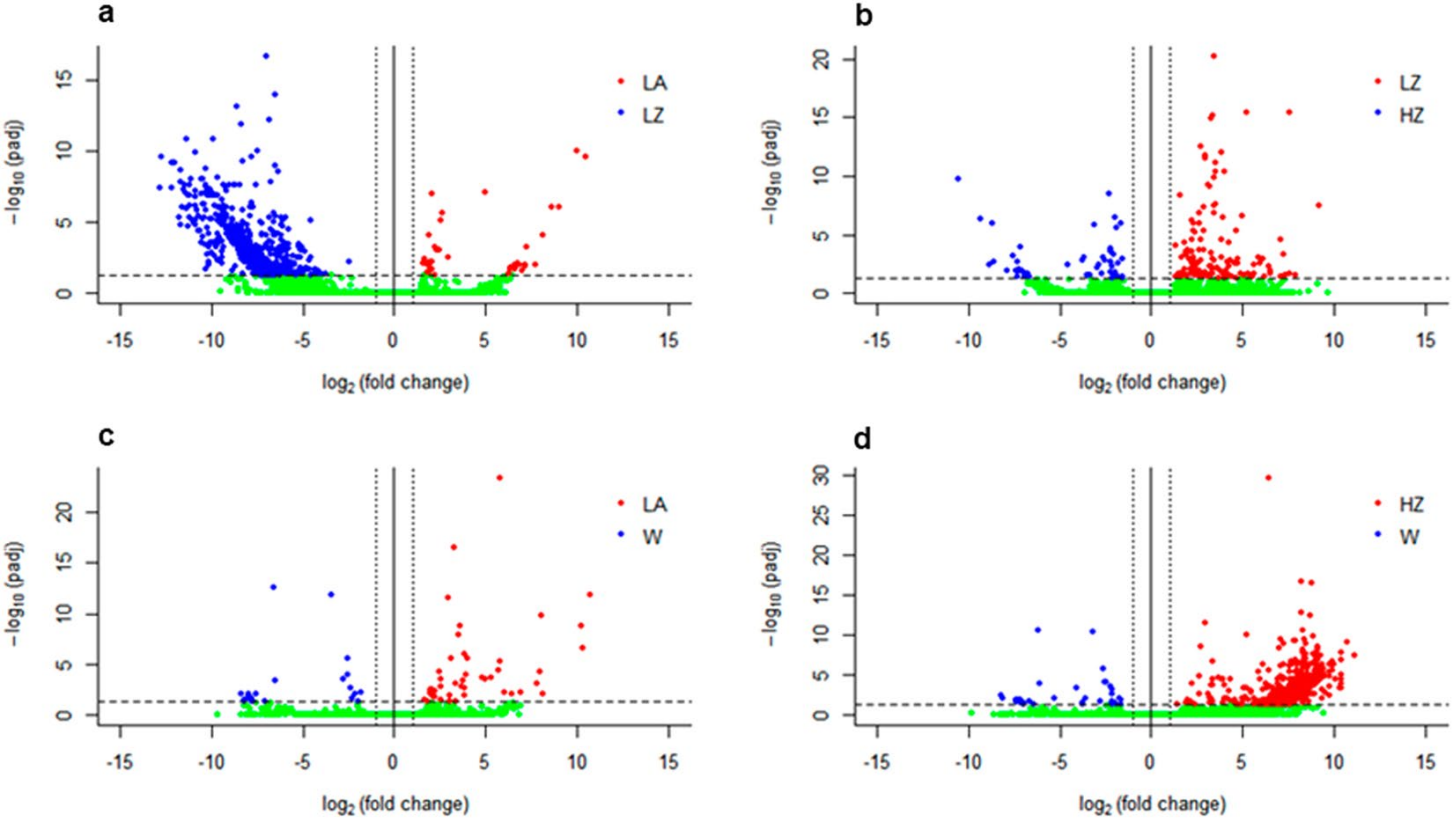
Volcano plot displaying differential expressed genes between the paralarvae culture groups: (LA) feed on *Artemia*, (LZ) feed on zoea, (W) wild paralarvae and (HZ) high temperature. The y-axis corresponds to the mean expression value of log_10_ (q-value), and the x-axis displays the log_2_ fold change value. Each dot represents one gene. The red dots represent the up-regulated expressed genes (FDR < 0.05 and fold-change > 1) in each contrast. Blue dots represent the down-regulated expressed genes. Green dots represent no differential expression genes.

The diet comparison (LA vs LZ) reported 673 significantly regulated genes. Among them, 635 (94.35%) were upregulated in zoea fed (LZ) paralarvae compared to 38 (5.65%) upregulated genes found in *Artemia* (LA) group (Fig. 5A). The functional enrichment analysis for LZ group (Table 4) reported GO terms related with lipid and carbohydrate metabolism (63 up-regulated genes related to GO terms GO:0042632, GO:0009395, GO:0005975, GO:0006094), immune response (22 up-regulated genes related to GO terms GO:0006955, GO:0071346, GO:0051092, GO:0070498, GO:0038061) and gene expression regulatory related terms (i.e. “methylation” (GO:0032259), 9 up-regulated genes) as enriched terms. In terms of stress, at least 33 up-regulated genes in LZ were associated to the term “response to stress” (GO:0006950).

**Table 4:** GO enrichment analysis, overview of the most relevant results.

Comparison	Samples	GO ID	Specific term	# genes
LA vs LZ	LZ	GO:0009062	fatty acid catabolic process	7*
		GO:0042632	cholesterol homeostasis	5*
		GO:0009395	phospholipid catabolic process	4*
		GO:0005975	carbohydrate metabolic process	37*
		GO:0006094	gluconeogenesis	10***
		GO:0006955	immune response	39*
		GO:0071346	cellular response to interferon-gamma	5**
		GO:0051092	positive regulation of NF-kappaB transcription factor activity	6*
		GO:0070498	interleukin-1-mediated signaling pathway	4*
		GO:0038061	NIK/NF-kappaB signaling	6*
		GO:0032259	methylation	9*
		GO:0034599	cellular response to oxidative stress	12**
		GO:1902175	regulation of oxidative stress-induced intrinsic apoptotic signaling pathway	3*
LZ vs HZ	LZ	GO:0046500	S-adenosylmethionine metabolic process	3***
		GO:0006695	cholesterol biosynthetic process	4***
		GO:0006577	amino-acid betaine metabolic process	3***
	HZ	GO:0006589	octopamine biosynthetic process	2***
		GO:0042420	dopamine catabolic process	2***
		GO:0042421	norepinephrine biosynthetic process	2***
LA vs W	LA	GO: 0019377	glycolipid catabolic process	2*
		GO:0007586	digestion	2*
		GO:0090287	regulation of cellular response to growth factor stimulus	1*
		GO:0006030	chitin metabolic process	4***
		GO:0055072	iron homeostasis	3**
LZ vs W	LZ	GO:0014012	peripheral nervous system axon regeneration	4***
		GO:0008652	cellular amino acid biosynthetic process	10*
		GO:0015991	ATP hydrolysis coupled proton transport	11***
		GO:0051603	proteolysis involved in cellular protein catabolic process	36***
		GO:0034440	lipid oxidation	8***
		GO:0055114	oxidation-reduction process	41**
		GO:0007586	digestion	7**
		GO:0006955	immune response	42**
		GO:0000302	response to reactive oxygen species	9**

The level of significance (Fisher’s exact test) is represented by: * to p < 0.05, **p < 0.005 and *** to p < 0.001. p < 0.05 was used as cut-off. Complete tables for each comparison can be found in supplementary data, including detailed p-values (Sup. Table 2). (LA) feed on *Artemia*, (LZ) feed on zoea, (W) wild paralarvae and (HZ) high temperature.

A total of 168 genes were regulated when comparing the effect of temperature (LZ vs HZ) (Fig. 5B). 126 corresponded to up-regulated genes in LZ (75%) and 42 in HZ (25%). The LZ up-regulated genes showed the enrichment of GO terms related to metabolism (GO:0046500, GO:0006695, GO:0006577). For the HZ up-regulated genes the enriched GOs were “octopamine biosynthetic process” (GO:0006589) 2 genes, “dopamine catabolic process” (GO:0042420) 2 genes and “norepinephrine biosynthetic process” (GO:0042421) 2 genes (Table 4).

The *Artemia* feeding - wild animals comparison (LA vs W) reported the lowest number DGE, since a total of 64 genes were up-regulated (Fig. 5C): 44 (68.75%) corresponds to paralarve fed on *Artemia* and 20 (31.25%) were up-regulated in wild individuals. On the contrary, paralarvae co-fed on zoea compared to wild paralarvae reported 693 up-regulated genes (Fig. 5D) which mainly correspond to up-regulated genes on the LZ group, 663 genes (95.67%). Enriched GOs derived from *Artemia* up-regulated genes (LA in LA vs W comparison) are related to more intense catabolism and growth, as expected when individuals without food restrictions are compared to wild ones (GO: 0019377, GO:0007586, GO:0090287). Concerning co-feeding - wild comparison (LZ vs W), >95% of the regulated genes were over-expressed in co-feeding group. The overview of the enriched GOs associated to co-feeding diet showed enriched terms related mainly to growth and development (14 up-regulated genes related to GO terms GO:0014012 and GO:0008652); energy metabolism (103 up-regulated genes related to GO terms GO:0015991, GO:0051603, GO:0034440, GO:0055114, GO:0007586); and immune and stress responses (51 up-regulated genes related to GO terms GO:0006955 and GO:0000302).

A complete report of DGE and GO can be found as supplementary data (Sup. Table 1 and Sup. Table 2).

### Gene expression profile on selected genes

**Figure 6:**
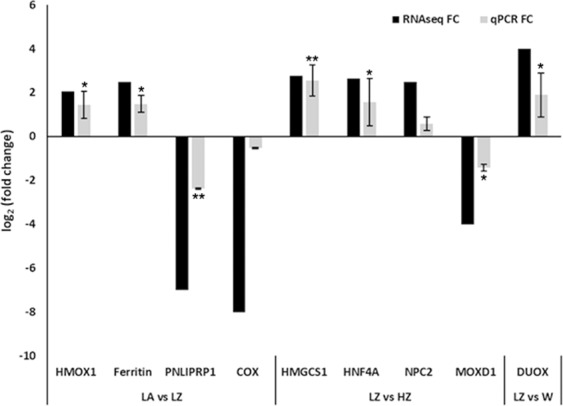
Expression variation of selected genes based on RNA-seq and RT-qPCR data. For 9 selected genes both technical approaches showed the same tendency and validate the transcriptomic analysis. The level of significance (paired Student’s t-test) is represented by: * to p < 0.05, **p < 0.005 and *** to p < 0.001. (LA feed on *Artemia*, (LZ) feed on zoea, (W) wild paralarvae and (HZ) high temperature.

Figure 6 shows the profile of gene expression on nine selected genes related to metabolic pathways including lipid metabolism and immune response. Gene expression results by RT-qPCR showed the tendency observed in RNA-seq results corroborating the success of the performed DGE analysis based on transcriptomic data.

### Identification of differentially expressed proteins by SWATH

**Figure 7:**
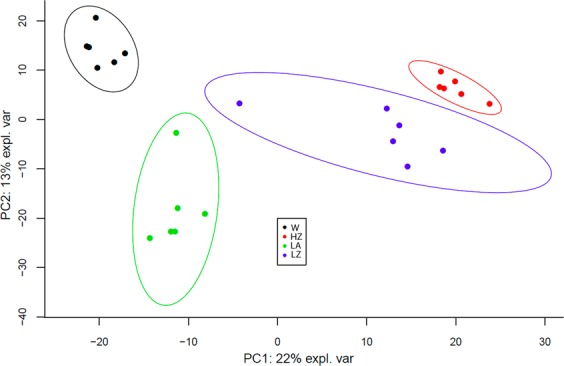
PCA plot of proteomic data of the *O*. *vulgaris* paralarvae individuals under study. Representation of the overall similarity, based on their protein expression profiles, between the 24 sequenced individuals categorized in 4 groups: feed on *Artemia* (LA), feed on zoea (LZ), wild paralarvae (W) and high temperature (HZ).

A total of 1,313 proteins were identified by SWATH using the protein database of the potential ORFs (open reading frame) obtained running the TransDecoder pipeline on our de novo assembled transcriptome. The Elactic Net regression model applied to the different comparison between the experimental groups under study made possible to found which proteins are the best to separate the groups. The different diet comparisons performed resulted in 66 proteins affected in their expression in LA vs LZ group, 48 proteins in LA vs W group and 56 proteins in LZ vs W group. Whereas temperature changed the expression of 44 proteins (LZ vs HZ) (Fig. 7). These proteins show differential expression related with diet or temperature which allowed to separate the experimental groups. PC1 summarizes approximately 22% of the variance and clearly makes possible to distinguish between diets. However, at protein level differences caused by temperature are less evident.

**Figure 8:**
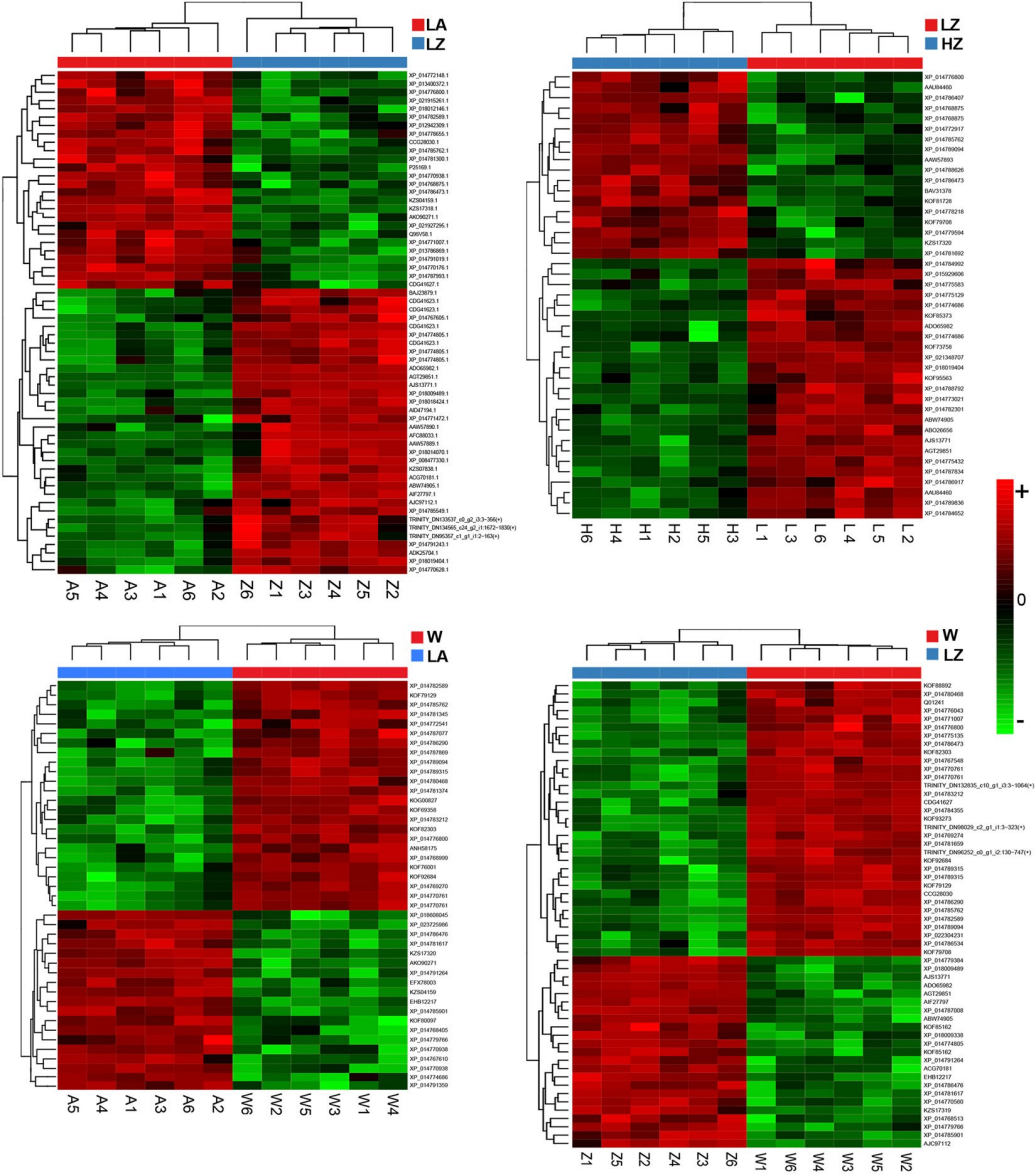
Individual heatmap representations of the differential expressed proteins in *O*. *vulgaris* paralarvae identified by SWATH. **LAvsLZ**. Heatmap of differential expressed proteins from LA vs LZ group (columns as A and Z respectively). **LZvsHZ**. Heatmap of differential expressed proteins from LZ vs HZ group (columns as L and H respectively). **WvsLA**. Heatmap of differential expressed proteins from W vs LA group (columns W and A respectively). **WvsLZ**. Heatmap of differential expressed proteins from W vs LZ group (columns W and Z respectively). Low to high expression is shown by a gradation color from red to green. On the right, the Uniprot Accession number of proteins is shown (for a more detailed list of proteins differentially expressed see Supplementary Table 3). (LA) feed on *Artemia*, (LZ) feed on zoea, (W) wild paralarvae and (HZ) high temperature.

The representation of the differentially expressed proteins in a heatmap splits the samples into clusters according with the condition of the individual paralarvae (Fig. 8). More in detail, the regression model shows up-regulation of proteins involved in glycolysis such as transketolase (XP_014768875) and opine dehydrogenase involved also in redox process (XP_014786407), lipid transport and amino acid transport under high temperature condition. Paralarvae grow in low temperature show up-regulation of different cathepsins and proteins related to lipid metabolism (XP_015929606; XP_014784992) among others.

Diet comparison reported up-regulated proteins involved in glycolysis process in paralarvae co-fed on zoea such as GADPH (XP_014791243.1; ADK25704.1) and transketolase among others. Additionally, an overexpression of ferritin in *Artemia* feed paralarvae is observed (XP_014770938.1; XP_014770938; XP_014770938).

A complete report of differential protein expression analysis can be found as supplementary data (Sup. Table 3).

## Discussion

The use of “omic” techniques has offered the opportunity to study non-model organisms at a massive level. This fact was also reflected on cephalopod research as these studies became more frequent in the last five years. In the present study, we have obtained for first time a transcriptome of *O*. *vulgaris* paralarvae with a 23% of annotation success which is similar to the previously published transcriptomes in cephalopods^27,47–49^. The next-generation proteomic SWATH-MS technology was also applied in the present work allowed us the successful identification of a total of 1,313 proteins after building an ad-hoc library from the transcriptome. Proteomic analysis was recently published applied to adult cephalopods by 2D MS-MS^50^ and paralarvae stages using 2D-DIGE^51^ approaches with successful results. However, this is the first time that a next generation label-free quantification proteomic approach has been performed on a cephalopod species. The robustness of our data has been proved by in-silico quality checks and also by biological analysis through the verification of DEG profiles by RT-qPCR. The PCA analysis plot showed how the individual samples grouped concordantly whit their experimental groups based in their transcriptomic profile. The fact that the quality checks and contrasted analysis corroborated our results support their inclusion in public databases to increase the information available to date in this species.

In relation to culturing success, diet was described to be the main factor that can determine a proper paralarvae development^1,7^. Our results showed that paralarvae fed exclusively with *Artemia* experienced lower growth and survival than those co-fed on zoea, artemia based culture has to be ended after 15 dph while the paralarvae in co-feeding live for 20 dph. These results are in concordance with previous assays using the same dietary design^20^. *Artemia* is, indeed, broadly used in farming of different marine organisms which need to fed on live preys^52^. However, *Artemia* based diets have shown some deficiencies, especially in terms of providing an adequate lipid profile^53,54^. The use of enriched treatments or the addition of zoea have been shown to balance the diet improving its nutritional value^53^.

The differential expression analysis between diets showed that genes up-regulated in paralarve co-fed on zoea encoded for a number of lipases and lipid transporters including the short-chain specific acyl-CoA dehydrogenase, NPC intracellular cholesterol transporter 1, low-density lipoprotein receptor and pancreatic lipase-related protein 2. However, analysis of enriched GO did not show any signal of lipid metabolism in individuals fed solely with *Artemia*. Our results might indicate that co-feeding with zoea could be associated to the observed changes in the gene expression profile due to improved balance of the lipid content^53,54^. It was demonstrated that zoea supplies the proportion of essential long chain polyunsaturated fatty acids (LC-PUFA), such as arachinodic (ARA), eicosapentaenoic acid (EPA) and docosahexaenoic acid (DHA)^55,56^ which in turns modify the profile of those genes involved in their metabolism. Indeed, several genes related to LC-PUFA such as stearoyl-CoA desaturase-5, acyl-CoA dehydrogenases, long-chain fatty acid transport protein-4, glycerol-3-phosphate acyltransferase-4 and acyl-coenzyme A thioesterase 3 appeared up-regulated in paralarvae co-fed on zoea. Interestingly, *Artemia* has a higher percentage of lipids in their composition compared to zoea^54^. However, this organism has been described to perform a rapid bioconversion of DHA from polar to neutral fraction^57,58^. In contrast, a study on reared *O*. *vulgaris* paralarvae showed most DHA is in polar fraction such as n-6 and n-3 HUFA after 10–30 days of feeding^59^. The fraction in which the fatty acids are located in the *Artemia* might make them unavailable to be metabolized by octopus paralarvae and this could be the reason to have not found DEG related to lipid metabolism. Whether an excess of lipids could also cause problems to the organism remains unknown. A delay in developmental of intestinal structures were described by histology in paralarvae fed exclusively with *Artemia*^28^. Our data showed less expression of the gene encoding for lysophospholipid acyltransferase in paralarvae fed on *Artemia*. Deficiencies on the activity of this gene have been related to intestinal damage^60^ which could also explain the poor performance paralarvae fed on *Artemia* in terms of growth.

An increased lipid metabolism seems to be one of the main differences in co-feeding with zoea and might also indirectly influence on the survival rate and growth. Indeed, long chain polyunsaturated fatty acids are basic components of membranes^61^ and were demonstrated to have a strong impact in the proper functioning of immune and neuronal activities^61^. It was demonstrated that the profile of fatty acids supplied by the diet can modulate cell immune functions through membrane alterations^62^ regulating signal transduction pathways and lipid mediators. A direct relationship between the synthesis of eicosanoids and the antibacterial response of haemocytes has been demonstrated in bivalves^63^. This finding supports the benefit of supplying zoea in the diet especially at paralarvae stage where susceptibility to aggressors might be critical. Lipid metabolism genes related to neuronal and vision development were also upregulated in paralarvae co-fed on zoea including the 2-hydroxyacylsphingosine 1-beta-galactosyltransferase implicated in the metabolism of galactocerebroside^64^ and glycerol 3 phosphate dehydrogenase and long-chain fatty acid transport protein 4, involved in vision development. Co-feeding with zoea seems also to stimulate the lipid metabolism and a correct lipid function might be crucial during early development stages^65^. We hypothesize that higher differences related to lipid metabolism induced by the diet should occur at later stages in the life cycle since basic metabolic requirements might be partially covered by yolk reserves at early post-hatching period, until 10 dph^19,66,67^. Therefore, it would be of high interest to study the development of octopus paralavare co-fed on zoea at later stages and decipher the benefits of this dietary system at medium and long-term.

Genes related to choline metabolism, such as ectonucleotide pyrophosphatase/phosphodiesterase family member 6 (ENPP6)^68^ and phosphatidate phosphatase (LPIN2) were up-regulated in paralarvae co-fed on zoea. Choline and its related metabolites assure the structural integrity and signaling functions of cell membranes, and it is involved in the synthesis of essential phospholipid components like phosphatidylcholine (PC) and sphingomyelin (SM). Protein encoded by ENPP6 gene was described to hydrolyze choline-containing compounds to ensure the availability of choline to the cell. On the other hand, LPIN2 gene was related to phosphatidlycholine biosynthesis. Less expression of these genes in paralarvae fed exclusively on *Artemia* could indicate choline and PC deficiencies derived by the lack of zoea co-fed, which could result in hepatic, renal, pancreatic, memory and growth disorders as has been shown in studies performed in humans^69^. Choline and PC levels can be also increased in culturing species by enriching *Artemia* as was demonstrated in some fishes, producing a significant increase in larvae growth and survival^70^. This approach deserves to be also tested in octopus paralarvae to maintain a proper level of these metabolites and evaluate its effect on culture performance.

Regarding energy obtaining, it is considered that cephalopod showed a more intense use of proteins and carbohydrates compared to lipids^71–75^. However, the accumulation of lipids in the digestive gland and their mobilization in some situations such as starvation might indicate a relative dependence on lipid catabolism^7,71^. A study carried out on cuttlefish showed that the global capacity for fatty acid oxidation was low but also might be tissue specific and conditioned by specific factors^71^.

In this context, the most highly up-regulated gene found in paralarvae co-fed on zoea encoded for gamma-butyrobetaine dioxygenase which is involved in L-carnitine biosynthesis. L-carnitine is an essential element of lipid metabolism which its main function is to introduce lipids into the mitochondria to feed the beta-oxidative process. This route occurred under the control of the carnitine O-acetyltransferase also found up-regulated in paralarvae co-fed on zoea. Since L-carnitine shows relative low levels in *Artemia*, the catabolism of lipids through the beta-oxidation process might have suffered a bottle-neck in paralarvae fed exclusively on *Artemia*^76^. The role of L-carnitine in the diet of farming species has been well studied in fishes^77^. Different dietary assays have demonstrated the positive impact of supplying L-carnitine enriched *Artemia* over the aquaculture performance i.e. *Trachinotus ovatus* and tilapias^78,79^. However, the enrichment treatment did not have a clear positive effect in zebrafish^80^. Regarding cephalopods, Matsubara *et al*.^81^ analyzed the impact of the use of *Artemia* on the nutrient levels of *O*. *sinensis*, showing an important depletion in the L-carnitine levels from hatching until 20–40 dph, when the L-carnitine level drastically rose and was not detected. Therefore, the overexpression of beta-oxidation and carnitine related genes (long-chain acyl-CoA dehydrogenase and short-chain acyl-CoA dehydrogenase) on paralarvae co-fed on zoea suggest that the lipid catabolic route could be also activated to obtain energy. Whether this route is activated due to the L-carnitine levels or due to different lipid composition compared to *Artemia* diet remains unknown. However, it should be clarified to improve the dietary formulations.

The KEGG annotation showed the carbohydrate metabolism as one of the most represented metabolic pathway in paralarvae co-fed on zoea compared to *Artemia* diet suggesting a positive effect of this dietary complement. Indeed, genes directly related to gluconeogenesis were found up-regulated such as the glucose-6-phosphatase (G6P) which is the direct responsible for generating free glucose. Less expression of the gene coding for G6P after Artemia feeding make us question if this diet might supply the necessary substrates to produce glucose through gluconeogenesis, which could be crucial to cover the energy demand of the paralarvae^71^. Our results were also confirmed by the proteomic analysis. Indeed, diet comparison by SWATH-MS reported different over-expressed proteins related to the glycolysis process in paralarvae co-fed on zoea such as glyceraldehyde-3-phosphate dehydrogenase (GADPH), and transketolase, which is also in concordance with the up-regulation of hexoquinase found at transcriptomic level. Few information is available to date regarding the impact of different diets on octopus paralarvae carbohydrate metabolism. To our knowledge this is the first study reporting that some deficiencies related to glucose synthesis might be occurring in paralarvae exclusively fed on *Artemia*. Since carbohydrates constitute the main sources of fuel in cephalopods^71^ under regular conditions, the study of their requirements should be stated in detail to formulate a proper diet to obtain good morphometric parameters in culturing animals.

The gene betaine-homocysteine S-methyltransferase 1 (BHMT) was one of the most expressed in paralarvae co-fed on zoea compared to the *Artemia* diet. BHMT is involved in amino acid metabolism and has an important role in homocysteine metabolism by catalyzing the transfer of a methyl group from betaine to homocysteine to produce methionine^82^. It might also be related to choline metabolisms through the synthesis of betaine by choline oxidation. BHMT might operate as a key enzyme since methionine is also the precursor of the major methyl donor of most of the biological methylation reactions^83^. Methionine levels on *Artemia* and zoea are quite similar^10^. However, as stated by our results there might be an alternative pathway to obtain this amino acid through the use of choline that might be augmented in paralarvae co-fed on zoea. Annotated transcripts related to alanine transport and mobilization and cysteine metabolism were also upregulated in co-fed animals and less regulated after supplying only *Artemia*. These genes included the alanine–tRNA ligase (AARS), the β-ureidopropionase (UPB1) and the cystathionine γ-lyase (CTH). Cysteine is a limiting element in the synthesis of glutathione which has antioxidant activity to protect the eye crystalline from reactive oxidative species^84^. Cysteine requirements could be supplied by the diet or by the conversion from methionine trough the activity of the CTH^85^. However, we have seen indicators of a potential deficiency of methionine in individuals fed on *Artemia* when they are compared with the co-feeding diet. All this information has lead us to hypothesize that the amino acid metabolism might be altered in animals fed exclusively with *Artemia*. This statement was also supported by the fact that proteins related to the obtaining of energy through leucine, isoleucine and valine catalysis (such as the 2-oxoisovalerate dehydrogenase identified also by SWATH-MS method) was up-regulated in *Artemia* fed paralarvae which could be derived from a scarceness of their main energy resources (carbohydrates).

At structural level through lipid conformation and metabolism and at energetic level by the use of carbohydrates, lipids and amino acids, the co-feeding with zoea seems to contribute to a better general organism state compared to the feeding exclusively on *Artemia*. Indeed, the good condition of the paralarvae might be maintained even when they are cultured at higher temperature.

In this study, when temperature effect was analyzed only using the co-feeding diet in order to avoid its influence, we observed a higher growth in octopus paralarvae culture at high temperature which could be a consequence of an increment at metabolic rate. Within the up-regulated genes in high temperature group we have found two genes coding for vitellogenins. Although vitellogenins have been associated mainly with reproductive processes, heat acclimation was recently described among the diverse functions of this protein family in honeybee larvae^86^. It is well known that the process of acclimation induces energy metabolism regulations and antioxidant responses. Genes such as ADP/ATP translocase 1 and NADH-ubiquinone oxidoreductase chain 4, related with the respiratory chain were up-regulated in high temperature cultured paralarvae (H, 19 ± 1 °C) which suggest that the energy requirements increased compared to low temperature reared animals (L, 15 ± 1 °C). The gene coding for DBH-like monooxygenase protein 1 which play a role in octopamine metabolism was also up-regulated after high temperature culture^87,88^. Octopamine was correlated with periods of extended activity and recovery from increased energy demand in other invertebrates, and this fact correlates with our findings suggesting an increased energy metabolism related to the predator behavior of the paralarvae cultured at higher temperature.

Genes related with the catabolism of carbohydrates, the main fuel source to support this high metabolic rate, were also up regulated such as the malate dehydrogenase 1B and transaldolase genes. The protein opine dehydrogenase was overexpressed which might ensure a proper flux trough glycolysis and a constant supply of ATP by maintaining the NADH/NAD+ ratio under swimming and predator activities^89^. Our proteomic analysis also reported the over-expression of transketolase under high temperature condition. Transketolase and transaldolase enable the interaction between the pentose phosphate pathway of carbohydrate transformation and glycolysis. These proteins were involved in a mechanism which favors the cell adaptation to its metabolic needs, controlling the synthesis of a number of coenzymes, vitamins, and precursors for nucleotide synthesis^90^. Several genes related with antioxidant activities were up-regulated under high temperature conditions, such as the vitellogenins mentioned above, apolipoprotein D and S-crystallin. In addition to antioxidant activity, apolipoprotein D has been related also with the reduction of lipid peroxidation trough interfering in eicosanoid metabolism which could explain the absence of lipid metabolism elements within the over-expressed genes and proteins under high temperature conditions^91^. Although S-crystallin was related with starving periods and eye development processes in *O*. *vulgaris* paralarvae^51^, it might be also involved in antioxidant related responses as the protein contains a glutathione-S-transferase domain which was described to be directly involved in reactive oxygen species elimination^92,93^.

All together these results might suggest that a more demanding energetic metabolism is occurring under 20 ± 1 °C vs 15 ± 1 °C and also the co-feeding diet supplied the required nutrients to carry out this enhanced activity.

The overall analysis of DEG on wild paralarvae compared to paralarvae fed on *Artemia* or co-fed on zoea showed higher similarities between wild and *Artemia* fed paralarvae since the profile of gene expression showed a greater number of genes similarly regulated. However, comparison between wild and zoea fed animals revealed a higher number of genes up-regulated under co-feeding conditions.

Enriched GOs analysis and the differentially expressed genes seem to indicate that a more intense catabolism, growth and development might be occurring in reared paralarvae compared to wild animals. Indeed, terms related to glycolipid catabolism and digestion were less represented in wild paralarvae compared to *Artemia* fed paralarvae. On the other hand, nervous system development, amino acid biosynthesis, energy metabolism, digestion or immune response related genes were up regulated in paralarvae co-fed on zoea. It is interesting to observe more similarities between feeding from the environment and *Artemia* diet at DGE level, considering that co-feeding with zoea might be more similar to the diet from the environment mainly composed by decapods^94^. Many questions arise from these results regarding the status of the wild paralarvae sampled in our study. It could happen that wild paralarvae were in good conditions to grow up to well-developed animals. In this case, their metabolism might be correctly operating however it seems decelerated compared to paralarvae reared under captivity conditions with no limitation on food and without predator pressure. On the other hand, it could also occur that wild paralarvae were in bad conditions to reach adulthood. Considering the reproductive strategy of this species it might be plausible to suppose that most of the progeny would not successfully end their development due to, among others, malformations that might limit movements and efficient feeding^84^. Therefore, the probability to have captured the proportion of those paralarvae that would reach adulthood might be very low. Our results on wild animals might be carefully treated before extracting conclusions and more detailed studies should be performed in a time series to check the evolution of their transcriptomic profile.

The number of up-regulated genes and enriched GO terms related to some metallic ions such as oligo elements acting as cofactor of enzymes was a remarkable output of our transcriptomic analysis. Genes related to copper were up-regulated in paralarvae co-fed on zoea compared to *Artemia* diet (low temperature conditions) and wild animals. On the other hand, reared animals compared to wild paralarvae showed an overexpression of genes related to iron. Unlike other aquatic organisms with hemoglobin, the protein that transports oxygen in *O*. *vulgaris* is hemocyanin, a metalloprotein shared by some invertebrates which contain copper as oxygen binds. This fact places copper as an essential oligo element for the oxygenation and therefore survival of the paralarvae. Indeed, the amount of copper obtained from *Artemia* has been proposed as a limiting element to growth in reared animals^95^. Villanueva *et al*.^9^ studied the presence of oligo elements in different stages of *O*. *vulgaris* and also in the main live prey *Artemia* and zoea concluding that zoea contains approximately ten times more copper than *Artemia* and this one possess near to three times more iron than paralarvae. Paralarvae fed on *Artemia* over-expressed transcripts encoding for heme oxygenase 1, a protein which cleaves heme rings presents in the *Artemia* hemoglobin. This degradation process might be responsible of an excess of intra cellular iron, that could be a source of toxicity if the paralarvae is not able to reduce it. At 10 dph an overexpression of ferritin was observed at transcriptomic and proteomic level in *Artemia* fed paralarvae. However, this could be an insight of a potential continuous storage of iron until reach toxic levels. Putative disorders caused by an excess of iron should be particularly checked in future studies on diets containing *Artemia*. Indeed, recent reports highlighted the degenerative process caused by iron excess through a mechanism named ferroptosis^96^, a programmed cell death dependent on iron. However, supplying zoea to the diet might contribute to balance and store this excess of iron.

Previous studies confirmed that the demethylation along *O*. *vulgaris* early development was directly driven by the age of the paralarvae, and the use of a diet enriched with long chain poly-unsaturated fatty acids to emulate decapods produced an acceleration of the global process of demethylation^97,98^. The current results agreed with these conclusions since we detected an overexpression in trasncripts related to methylation, such as putative methyltransferase, inactive histone-lysine N-methyltransferase 2E and lycine N-methyltransferase, in paralarvae co-fed on zoea in comparison to *Artemia* diet and wild animals. Temperature is also a factor related to changes in the DNA methylation of marine organisms, however in our study temperature did not seem to show any effect over the DNA methylation, at least for 10 dph paralarvae.

Proper diet formulation and abiotic factors like temperature have been long recognized as a requirement for welfare and health, since nutrients may alter immune responses by acting directly on the immune cells or indirectly through metabolic, neurological or endocrine pathways^99,100^. The fact that genes associated to GO terms related to immunity and defenses were less expressed in paralarvae fed on *Artemia* lead us to hypothesize that these paralarvae could have a lower basal level of immune related genes compared to co-fed animals with zoea. It would be of high interest to study under experimental infections if any benefit in terms of defense is occurring against aggressors after co-feeding with zoeae. In relation to this immune activity, differentially expressed terms associated to response to oxidative stress were also detected in co-fed animals. This response might be occurring as an intrinsic consequence to balance the cell damage associated to oxidative related defenses.

In contrast, the temperature of culture did not seem to have a direct impact on the immune response, only five genes related to immune response were overexpressed in paralarvae culture at higher temperature. However, when wild individuals were compared to reared animals, the latter showed a higher immune system activity. Therefore, our results might suggest that diet could have a higher impact on immune capacity of the paralarvae than other abiotic factors.

Through the addition of spider crab zoeae, paralarvae have received a balanced lipid profile incorporating also several oligoelements which might have a big impact in their overall performance. Carbohydrate and amino acid metabolism, as well as immune and stress response were also more regulated after zoeae co-feeding, suggesting a better general condition to face aggressors and to obtain energy from different sources. Our results might indicate that culturing at relative high temperature (20–21 °C) could not trigger deleterious effect to paralarvae development on the assumption that nutritional needs are covered. However, this situation could be problematic if paralarvae are kept until they reach the extenuation threshold. Future studies should be performed beyond to 10 days to evaluate the growth of the paralarvae at this high energy rate demand.

Altogether, our results pointed out the importance of evaluating the nutritional strategy for *O*. *vulgaris* culture under captivity focusing on the development of enrichment protocols or through the inclusion of new preys, to provide a more equilibrated diet.

The comparative study of the transcriptomic and proteomic profiles of paralarvae reared under different conditions, focused on diet and temperature, has allowed the identification of genes and proteins that deserve to be further studied and tested as candidates to biomarkers of development and health. Our results offer novel overall information and formulate new hypothesis on paralarvae welfare at nutritional level that will open new opportunities to deepen in paralarve rearing requirements.

## Supplementary information

Dataset S1

Dataset S2

Dataset S3
